# Real‐world study on the characteristics, post‐nephrectomy journey, and outcomes of patients with early‐stage renal cell carcinoma based on risk groups

**DOI:** 10.1002/cam4.7247

**Published:** 2024-06-03

**Authors:** Jose A. Karam, Rituparna Bhattacharya, Adesuwa Ogbomo, Santosh Gautam, Rebekah Yu, Murali Sundaram, Kentaro Imai, Jatin Chhabra, Naomi B. Haas

**Affiliations:** ^1^ Department of Urology and Translational Molecular Pathology The University of Texas MD Anderson Cancer Center Houston Texas USA; ^2^ Merck & Co., Inc Rahway New Jersey USA; ^3^ ConcertAI, LLC Cambridge Massachusetts USA; ^4^ University of Pennsylvania Perelman School of Medicine Philadelphia Pennsylvania USA

**Keywords:** kidney neoplasms, neoplasm recurrence local, nephrectomy, retrospective studies, risk factors

## Abstract

**Objectives:**

To examine real‐world characteristics, journey, and outcomes among patients with locoregional, nonmetastatic renal cell carcinoma (RCC).

**Methods:**

A retrospective analysis of medical records from the ConcertAI Oncology Dataset was performed on adults in the United States with newly diagnosed nonmetastatic RCC between January 2012–December 2017 who received surgical treatment, and were followed until August 2021. Patients were stratified based on the risk of recurrence after nephrectomy. Recurrence rate and survival outcomes were assessed.

**Results:**

The cohort (*n* = 439) had a median age of 64 years, 66.1% were male, and 76.5% had clear‐cell histology. The median follow‐up time from nephrectomy was 39.3 months overall, 41.0 months for intermediate‐high‐risk patients (*n* = 377; 85.9%) and 24.1 months for high‐risk patients (*n* = 62; 14.1%). For intermediate‐high‐ and high‐risk patients, respectively, 68.4% and 56.5% had ≥1 medical oncologist visit after nephrectomy. Of 260 patients with documentation of postoperative imaging assessments, 72% were ordered by medical oncologists, and the median time from initial nephrectomy to the first scan was 110 days (intermediate‐high‐risk) and 51 days (high‐risk). Provider‐documented recurrence occurred in 223 (50.8%) patients, of whom 41.7% had ≥1 medical oncologist visit before the recurrence. Three‐year disease‐free survival (DFS), and overall survival rates were 49.4% and 80.8% (all patients): 27.7% and 64.7% (high‐risk); and 52.9% and 83.3% (intermediate‐high‐risk).

**Conclusions:**

Our study reports low DFS after nephrectomy for patients with intermediate‐high‐ and high‐risk RCC. Subsequent approval and use of new and newly approved adjuvant therapeutic options could potentially delay or prevent recurrence.

## 1 INTRODUCTION

An estimated 628,355 people living in the United States (US) have been diagnosed with renal cell carcinoma (RCC).^1^ In 2023, approximately 81,800 people, representing 4.2% of all new cancer cases, were diagnosed with renal cancers, a consistent increase of about 1% annually over the last 15 years.^1^, ^2^ Up to 66% of RCC diagnoses are made when the tumor is still localized, 16% of diagnoses occur when the disease is locally advanced, and 15% after distant metastasis, with 5‐year survival rates of 93%, 74%, and 17%, respectively.^1^ The majority (75–87%) of patients with RCC have clear‐cell histology (ccRCC), and the remainder have nonclear‐cell/other RCC.^3^ Sarcomatoid dedifferentiation occurs in about 4% of patients overall, and 20% of patients with metastatic RCC.^4^ ccRCC and sarcomatoid dedifferentiation are both associated with poorer outcomes.^4^, ^5^, ^6^

Surgical resection is the primary treatment approach for RCC.^7^, ^8^ The current NCCN Clinical Practice Guidelines in Oncology (NCCN Guidelines®) recommend surgery or ablation as treatment options, followed by imaging surveillance, for localized and locoregional RCC.^9^ Adjuvant pembrolizumab is a recommended treatment option for select patients with stage II–IV ccRCC as post‐nephrectomy recurrence rates range between 25 and 55% for these patients.^8^, ^9^, ^10^, ^11^, ^12^ Currently, adjuvant systemic therapies include the vascular endothelial growth factor receptor (VEGF‐R) inhibitor sunitinib and the PD‐1 inhibitor pembrolizumab,^9^ approved by the US Food and Drug Administration (FDA), based on disease‐free survival (DFS) results, in 2017 and 2021, respectively.^13^, ^14^ To date, only adjuvant pembrolizumab has shown improved overall survival (OS) compared to placebo.^8^, ^12^, ^15^

The wide range of recurrence rates reported in the literature, reflecting diverse populations and surgical procedures, makes it difficult to discern which patients are at greatest risk of recurrence. Because disease recurrence is correlated with increased mortality as well as substantial healthcare utilization (HCU),^16^, ^17^ and given the limited treatment options available, it is crucial to identify those at greatest risk of post‐nephrectomy recurrence. While HCU has been documented for patients receiving systemic treatment^18^, ^19^ and in the postrecurrence setting,^17^ very few US real‐world studies have examined the RCC patient journey in terms of frequency of visits and imaging post nephrectomy.^20^ Our objective was to understand the characteristics, journey, treatment patterns, and outcomes for patients with intermediate‐high‐ and high‐risk RCC undergoing nephrectomy, to inform future research priorities in the adjuvant setting.

## 2 METHODS

### 2.1 Study design and patients

This was a retrospective, observational study using electronic medical record (EMR) data. Eligible patients were adults (age ≥ 18 years) diagnosed with nonmetastatic RCC (stage I, II, III, or IV M0) between January 1, 2012 and December 31, 2017 to allow for the potential of a 3‐year follow‐up period. Patients were classified based on the American Joint Committee on Cancer TNM classification, 7th and 8th editions.^21^, ^22^ Patients were required to have intermediate‐high‐risk (T2/N0/M0 with grade 4 cells or sarcomatoid histology; or T3/N0/M0) or high‐risk (T4/N0/M0, or any T stage with N ≥ 1) RCC at initial diagnosis, and to have received surgical treatment for RCC prior to metastatic RCC diagnosis. Patients were followed from the date of initial diagnosis until the end of the record, death, or data collection cutoff (August 19, 2021), whichever occurred first.

This chart review study involving human participants was performed in accordance with the ethical standards of the institutional and/or national research committee and with the 1964 Helsinki Declaration and its later amendments or comparable ethical standards. This research was reviewed and approved by the Institutional Review Board of Advarra, Columbia, Maryland. This research study was conducted retrospectively from data obtained for clinical purposes. An IRB waiver of consent was granted from Advarra.

### 2.2 Data source

Data were obtained from the ConcertAI Oncology Dataset, a consolidated oncology EMR database available to ConcertAI through data sharing agreements with practices and other data providers, including principally community oncology practices, representing diverse practice locations, both rural and urban centers, within the US. In addition to structured data fields, the dataset consists of unstructured clinical information including provider progress notes and images containing relevant information, such as the date and type of disease recurrence, and pathology and radiology reports.

### 2.3 Patient characteristics and journey

Patient characteristics included demographics and disease state data, including staging, histology, tumor grade, metastatic sites, Eastern Cooperative Oncology Group (ECOG) performance status, and comorbidities. Characterization of the patient's journey was based on the timing and utilization of RCC‐related healthcare, including surgery, imaging, and oncology visits.

### 2.4 Clinical outcomes

**FIGURE A1:**
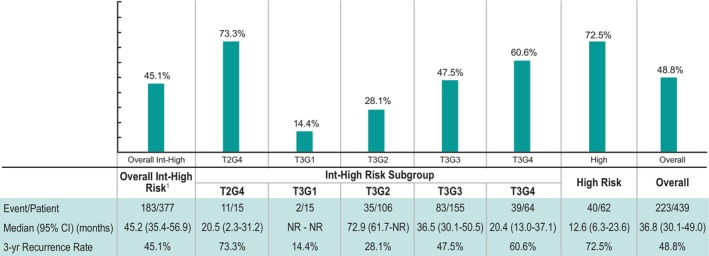
Kaplan–Meier analysis of 3‐year recurrence rate from initial nephrectomy following nonmetastatic RCC, overall and by risk category. ^1^Subgroups do not sum to 377; 22 patients had provider documentation of risk group but no documentation of tumor size (T) and/or tumor grade (G). CI, confidence interval; G, Fuhrman tumor grade; Int‐high, intermediate high risk;NR, not reached; RCC, renal cell carcinoma; T, tumor stage.

Real‐world clinical outcomes were time to recurrence, recurrence rate, DFS, and OS. Time to recurrence was defined as the time from initial nephrectomy to the first recurrence. The recurrence rate is the proportion of patients with documented recurrence event (results in Figure A1). DFS was defined as the time from initial nephrectomy to the first recurrence event or death.^23^ OS was defined as the time from initial nephrectomy until death. Patients without a terminal event for an endpoint were censored at the last encounter date/end of medical record.

### 2.5 Statistical methods

Demographic and clinical characteristics were evaluated descriptively for the overall population, and by risk cohorts. For outcomes analyses, the intermediate‐high‐risk cohort was further divided into subgroups based on tumor stage (T) and grade (G). We used Kaplan–Meier survival analysis methods to calculate 3‐year time to recurrence, DFS, and OS rates, and, in a subset of patients diagnosed between January 1, 2012 and December 31, 2015, we examined 5‐year DFS and OS rates. Cox proportional hazards regression analysis was used to evaluate risk categories associated with 5‐year OS.

## 3 RESULTS

### 3.1 Demographic characteristics by disease status

A total of 439 eligible patients with nonmetastatic RCC were identified and stratified into intermediate‐high‐risk (*n* = 377; 85.9%) and high‐risk (*n* = 62; 14.1%) cohorts. The median follow‐up time was 39.3 months, 41.0 months, and 24.1 months, overall and for the intermediate‐high‐ and high‐risk cohorts, respectively. The median (range) patient age was 64 (28–84) years, 66.1% of patients were male, and 80% were white (Table 1). Patient demographics were similar between risk cohorts, except the proportion of black/African American patients was larger in the high‐risk group (19.4% vs. 5.8%). 92.9% of patients were managed in the community (vs. academic) oncology setting.

**TABLE 1:** Baseline demographic and clinical characteristics, overall and by risk category.

Variable/statistic	Risk cohort		Overall (*N* = 439)
	Intermediate‐high (*n* = 377)	High (*n* = 62)	
Age (years), mean ± SD	63.6 ± 10.8	62.2 ± 12.2	63.4 ± 11.0
Age (years) median (range)	64 (28.0, 84.0)	64 (38.0, 82.0)	64 (28.0, 84.0)
Sex, *n* (%)			
Female	123 (32.6%)	26 (41.9%)	149 (33.9%)
Male	254 (67.4%)	36 (58.1%)	290 (66.1%)
Race, *n* (%)			
White	306 (81.2%)	45 (72.6%)	351 (80.0%)
Other/unknown	71 (18.8%)	17 (27.4%)	88 (20.0%)
Insurance category, *n* (%)			
Private only	94 (24.9%)	13 (21.0%)	107 (24.4%)
Medicare/Medicaid only	112 (29.7%)	20 (32.3%)	132 (30.1%)
Private and Medicare/Medicaid	14 (3.7%)	2 (3.2%)	16 (3.6%)
Unknown/undocumented	157 (41.6%)	27 (43.5%)	184 (41.9%)
Treatment setting, *n* (%)			
Academic	28 (7.4%)	3 (4.8%)	31 (7.1%)
Community	349 (92.6%)	59 (95.2%)	408 (92.9%)
Stage at initial diagnosis, *n* (%)			
II	20 (5.3%)	1 (1.6%)	21 (4.8%)
III	357 (94.7%)	51 (82.3%)	408 (92.9%)
IV	0 (0.0%)	10 (16.1%)	10 (2.3%)
Fuhrman grade at initial diagnosis, *n* (%)			
G1	11 (2.9%)	0 (0.0%)	11 (2.5%)
G2	97 (25.7%)	9 (14.5%)	106 (24.1%)
G3	131 (34.7%)	19 (30.6%)	150 (34.2%)
G4	72 (19.1%)	18 (29.0%)	90 (20.5%)
Undocumented	66 (17.5%)	16 (25.8%)	82 (18.7%)
Disease histology at initial diagnosis^a^, *n* (%)			
Clear cell	307 (81.4%)	29 (46.8%)	336 (76.5%)
Nonclear cell	35 (9.3%)	11 (17.7%)	46 (10.5%)
Others	35 (9.3%)	22 (35.5%)	57 (13.0%)
Sarcomatoid elements at initial diagnosis^b^, *n* (%)			
Yes	16 (4.2%)	10 (16.1%)	26 (5.9%)
No	361 (95.8%)	52 (83.9%)	413 (94.1%)
Comorbidities at initial diagnosis, *n* (%)			
Myocardial infarction	9 (2.4%)	3 (4.8%)	12 (2.7%)
Congestive heart failure	12 (3.2%)	1 (1.6%)	13 (3.0%)
Cerebrovascular disease	7 (1.9%)	4 (6.5%)	11 (2.5%)
Chronic obstructive pulmonary disease	13 (3.4%)	2 (3.2%)	15 (3.4%)
Diabetes	64 (17.0%)	10 (16.1%)	74 (16.9%)
Renal disease	34 (9.0%)	4 (6.5%)	38 (8.7%)
Weighted CCI, mean ± SD	0.5 ± 1	0.7 ± 1.3	0.6 ± 1
ECOG status, n (%)			
0–1	79 (21.0%)	20 (32.3%)	99 (22.6%)
≥2	7 (1.9%)	5 (8.1%)	12 (2.7%)
Undocumented	291 (77.2%)	37 (59.7%)	328 (74.7%)
Functional impairment^c^, *n* (% of undocumented ECOG)			
Yes	1 (0.3%)	0 (0.0%)	1 (0.3%)
No	280 (96.2%)	35 (94.6%)	315 (96.0%)
Undocumented	10 (3.4%)	2 (5.4%)	12 (3.7%)
Subtotal	291	37	328

Abbreviations: BMI, body mass index; CCI, Charlson Comorbidity Index; ECOG, Eastern Cooperative Oncology Group; G, Fuhrman tumor grade; HIV, human immunodeficiency virus; SD, standard deviation.

^a^ Disease histology classified into clear cell, nonclear cell, or others; please see Table A1 for details.

^b^ Disease histology classified into sarcomatoid versus nonsarcomatoid.

^c^ Based on chart review of patients for whom ECOG status was unknown.

Most patients (92.9%) were initially diagnosed with stage III disease, 78.8% had Fuhrman grade 2–4 tumors (grade was unavailable for 18.7%), 76.5% had clear‐cell histology (see Table A1 for histology classification), and the most common comorbidities were diabetes (16.9%) and renal disease (8.7%) (Table 1). ECOG performance status was undocumented for 74.7% of patients, while those with available data mostly had a score of 0 or 1 (22.6%). Among patients with undocumented ECOG, notes abstracted from medical records indicated that 96.0% had a nonimpaired performance status.

### 3.2 Patient journey

After receiving a diagnosis of RCC, 92.7% of patients received a radical nephrectomy (Table 2). Following nephrectomy, 66.7% of patients have a record of at least 1 follow‐up medical oncologist visit, which occurred after a median of 265.0 days. The follow‐up visit occurred sooner among high‐risk than intermediate‐high‐risk patients (median 64.0 vs. 351.5 days). High‐risk patients also had more follow‐up medical oncologist visits per year (median 4.7 vs. 2.3). Only 260 (59.2%) patients (60.2% of intermediate‐high‐ and 53.2% of high‐risk patients) had post‐nephrectomy imaging records available. 72.0% of imaging studies (*n* = 1783) were ordered by a medical oncologist and 19.1% by a urologist (Table 2). The median time from nephrectomy to the first follow‐up imaging assessment was 110.0 days for intermediate‐high‐ and 51.0 days for high‐risk patients, and the most common imaging modality was CT (56.0%) followed by MRI (18.9%) and PET (18.2%).

**TABLE 2:** Patient journey after nonmetastatic RCC diagnosis, overall and by risk category.

Variable/statistic^a^	Risk cohort		Overall (N = 439)
	Intermediate‐high (*n* = 377)	High (*n* = 62)	
Type of surgical resection, *n* (%)			
Partial nephrectomy	30 (8.0%)	2 (3.2%)	32 (7.3%)
Radical nephrectomy	347 (92.0%)	60 (96.8%)	407 (92.7%)
Follow‐up from initial nephrectomy to end of record/death, months			
Median	41.0	24.1	39.3
Oncology visit after initial nephrectomy, *n* (%)			
No	119 (31.6%)	27 (43.5%)	146 (33.3%)
Yes	258 (68.4%)	35 (56.5%)	293 (66.7%)
Time between initial nephrectomy and first oncologist visit, days			
N	258 (68.4%)	35 (56.5%)	293 (66.7%)
Median	351.5	64.0	265.0
Number of oncologist visits per year after initial nephrectomy			
N	258 (68.4%)	35 (56.5%)	293 (66.7%)
Median	2.3	4.7	2.6
Patients with records of imaging results after initial nephrectomy, *n* (%)			
No	150 (39.8%)	29 (46.8%)	179 (40.8%)
Yes	227 (60.2%)	33 (53.2%)	260 (59.2%)
Time between nephrectomy and first follow‐up imaging, days			
N	227	33	260
Median	110	51	105.5
Specialty of physician requesting for imaging, *n* (%)			
Oncology	119 (68.4%)	1165 (72.4%)	1284 (72.0%)
Urology	53 (30.5%)	288 (17.9%)	341 (19.1%)
Undocumented	2 (1.1%)	156 (9.7%)	158 (8.9%)
Type of imaging received, *n* (%)			
CT	216 (57.3%)	30 (48.4%)	246 (56.0%)
MRI	74 (19.6%)	9 (14.5%)	83 (18.9%)
PET	70 (18.6%)	10 (16.1%)	80 (18.2%)
Radioisotope scan	41 (10.9%)	9 (14.5%)	50 (11.4%)
Ultrasound	43 (11.4%)	6 (9.7%)	49 (11.2%)
X‐ray	60 (15.9%)	8 (12.9%)	68 (15.5%)
Other	8 (2.1%)	3 (4.8%)	11 (2.5%)
Number of imaging studies per year after initial nephrectomy			
*n* (patients)	227 (60.2%)	33 (53.2%)	260 (59.2%)
Median	2.3	3.1	2.4
Initiated systemic treatment within 90 days of initial nephrectomy, *n* (%)	21 (5.6%)	5 (8.1%)	26 (5.9%)

Abbreviations: CT, computed tomography; MRI, magnetic resonance imaging; PET, positron emission tomography; SD, standard deviation.

^a^ All patients included in analysis unless sample sizes (*n*) provided.

Only 26 (5.9%) patients initiated adjuvant systemic therapy within 90 days of the initial nephrectomy, including 21 (5.6%) intermediate‐high‐ and 5 (8.1%) high‐risk patients (Table 2). The most prescribed systemic agent was pazopanib (48.4%) followed by sunitinib (25.6%) (Table A2).

### 3.3 Outcomes after initial nephrectomy

About half (*n* = 223) of patients (48.5% intermediate‐high‐ and 64.5% high‐risk) had a documented recurrence after initial nephrectomy (Table 3). Of those who experienced a recurrence, 42% (93/223) attended a medical oncology visit between nephrectomy and recurrence (Table A3). Among patients with a recurrence, 85.2% had distant metastasis, most commonly in the lungs (12.6%) and liver (5.3%); the metastatic site was undocumented for 74.2% of patients (Table 3).

**TABLE 3:** Post‐nephrectomy recurrence, overall and by risk category.

Variable, *n* (%)	Risk cohort		Overall (*N* = 439)
	Intermediate‐high (*n* = 377)	High (*n* = 62)	
Experienced a recurrence	183 (48.5%)	40 (64.5%)	223 (50.8%)
Type of recurrence^a^			
Local/regional	25 (13.7%)	8 (20.0%)	33 (14.8%)
Distant metastasis	158 (86.3%)	32 (80.0%)	190 (85.2%)
Site of distant metastasis^b^			
Adrenals	4 (2.5%)	0	4 (2.1%)
Bone	6 (3.8%)	2 (6.3%)	8 (4.2%)
Brain	2 (1.3%)	0	2 (1.1%)
Lymph nodes	3 (1.6%)	5 (12.5%)	8 (3.6%)
Mediastinum	1 (<1%)	0	1 (<1%)
Omentum	1 (<1%)	0	1 (<1%)
Retroperitoneum	1 (<1%)	1 (3.1%)	2 (1.1%)
Soft tissue	1 (<1%)	1 (3.1%)	2 (1.1%)
Kidney	1 (<1%)	0	1 (<1%)
Liver	8 (5.1%)	2 (6.3%)	10 (5.3%)
Lung	20 (12.7%)	4 (12.5%)	24 (12.6%)
Pleura	1 (<1%)	0	1 (<1%)
Other	1 (<1%)	1 (3.1%)	2 (1.1%)
Undocumented	117 (74.1%)	24 (75.0%)	141 (74.2%)

^a^ Denominator based on numbers of patients who experienced a recurrence.

^b^ Denominator based on the numbers of patients with distant metastasis.

**FIGURE 1:**
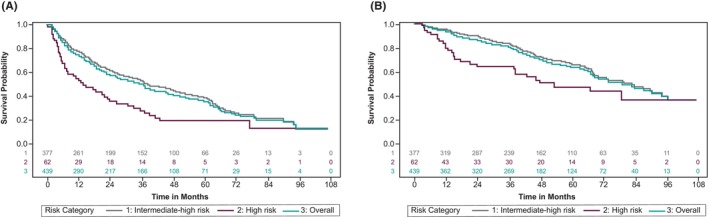
(A) Kaplan–Meier analysis of DFS from initial nephrectomy following nonmetastatic RCC, overall and by risk category (B) Kaplan–Meier analysis of OS, overall and by risk category. DFS, disease‐free survival; OS, overall survival; RCC, renal cell carcinoma.

**FIGURE 2:**
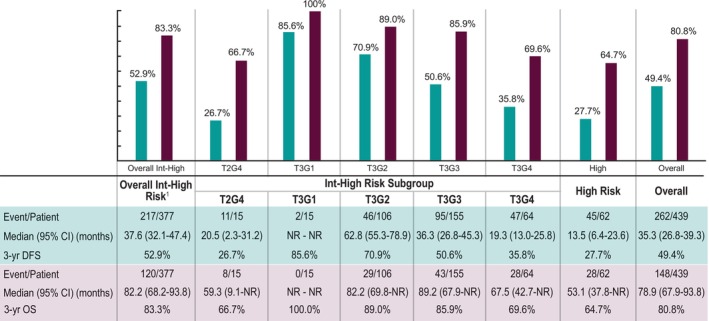
Kaplan–Meier analysis of 3‐year DFS and OS from initial nephrectomy following nonmetastatic RCC, overall and by risk category. ^1^Subgroups do not sum to 377, 22 patients had provider documentation of risk group but no documentation of tumor size (T) and/or tumor grade (G). CI, confidence interval; DFS, disease‐free survival; G, Fuhrman tumor grade; NR, not reached; OS, overall survival; RCC, renal cell carcinoma; T, tumor stage.

Kaplan–Meier plots for DFS and OS are presented in Figure 1. Among patients with at least 3 years of follow‐up (*n* = 439), median DFS and OS were 35.3 and 78.9 months, respectively. The 3‐year DFS rate was 49.4% and approximately 80% of patients were alive after 3 years (Figure 2). For high‐risk patients, the median DFS was 13.5 months, the median OS was 53.1 months, and the 3‐year DFS and OS rates were 27.7% and 64.7%, respectively. The corresponding figures for the intermediate‐high‐risk patients were 37.6 months median DFS, 82.2 months median OS with a 52.9% 3‐year DFS and 83.3% 3‐year OS rate. A breakdown by intermediate‐high subgroup can be seen in Figure 2.

**FIGURE 3:**
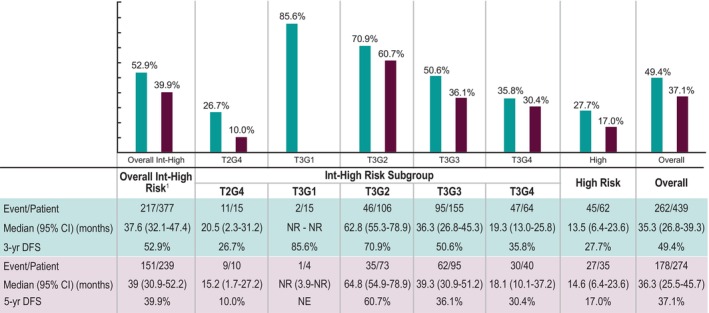
3‐Year and 5‐Year DFS from initial nephrectomy following nonmetastatic RCC, overall and by risk category. CI, confidence interval; DFS, disease‐free survival; Int‐high, intermediate high risk; NE, not evaluable; NR, not reached. RCC, renal cell carcinoma.

Among patients with at least 5 years of follow‐up (*n* = 239), the median 5‐year DFS was 36.3 months, ranging from 14.6 to 39.0 months for the high‐ and intermediate‐high‐risk groups. The 5‐year DFS rate was 37.1% overall, 17.0% and 39.9% for the high‐risk and intermediate‐high‐risk groups, respectively (Figure 3). A Cox regression analysis of 5‐year OS revealed that, after controlling for age, sex, race, histology, and ECOG, patients with a recurrence were 2.4 times more likely to die within 5 years of initial nephrectomy compared to patients without recurrence (Hazard ratio [HR] = 2.43; 95% confidence interval [CI]: 1.51–3.91). The only other factor significantly associated with 5‐year OS was increased age at initial RCC diagnosis (HR = 1.03; 95% CI: 1.01–1.05) (Table 4).

**TABLE 4:** Cox regression analysis for assessing factors associated with 5‐year OS following nephrectomy^a^.

Parameters	Estimate ± SE	*p*‐Value	Hazard Ratio (95% CI)
Recurrence (with vs. without)	0.887 ± 0.243	0.0003*	2.427 (1.508–3.907)
Risk category (high vs. intermediate‐high)	0.32 ± 0.305	0.2941	1.377 (0.758–2.503)
Age at initial diagnosis (continuous)	0.032 ± 0.01	0.0013*	1.033 (1.013–1.053)
Female versus male	0.076 ± 0.21	0.7157	1.079 (0.715–1.629)
Other/unknown versus White race	−0.438 ± 0.275	0.1107	0.645 (0.377–1.105)
Clear cell versus nonclear cell/other at initial diagnosis^b^	−0.032 ± 0.235	0.8932	0.969 (0.611–1.536)
ECOG status at initial diagnosis			
1 versus 0	0.43 ± 0.464	0.3542	1.537 (0.619–3.815)
≥2 versus 0	1.264 ± 0.806	0.1169	3.54 (0.729–17.189)
Undocumented versus 0	0.092 ± 0.355	0.7958	1.096 (0.546–2.200)

Abbreviations: CI, confidence interval; ECOG, Eastern Cooperative Oncology Group; OS, overall survival; SE, standard error.

^a^ Based on patients diagnosed through December 31, 2015, to allow 5 years of follow‐up prior to the data cutoff date, *n* = 239.

^b^ Please see Table A1 for details on classification.

* *p* < 0.05.

### 3.4 Outcomes by RCC histology: Clear cell versus nonclear cell/other

Three‐year recurrence rates were 46.4% and 56.1%, for ccRCC and non‐ccRCC/other, respectively (Table 5). Assessments of DFS and OS showed median DFS was 37.1 months for patients with ccRCC (*n* = 336) compared with 20.6 months for non‐ccRCC/other (*n* = 103), while median OS was 82.2 versus 72.8 months, respectively (Table 5). The 3‐year DFS rate was 51.3% for patients with ccRCC compared to 43.2% for patients with non‐ccRCC/other. The 3‐year OS rates were 82.4% and 75.7% for patients with ccRCC and non‐ccRCC/other, respectively.

**TABLE 5:** Kaplan–Meier analysis of time to recurrence, DFS, and OS from initial nephrectomy following nonmetastatic RCC, overall and by histology at initial diagnosis.

Variable/statistic	Histology^a^		Overall (*N* = 439)
	Clear cell (*n* = 336)	Nonclear cell/other^a^ (*n* = 103)	
Time to Recurrence			
Events, *n*	158	65	223
Median, months	44.9	21.5	36.8
3‐year recurrence rate, %	46.4%	56.1%	48.8%
DFS			
Events, *n*	192	70	262
Median, months	37.1	20.6	35.3
3‐year DFS, %	51.3%	43.2%	49.4%
OS			
Events, *n*	107	41	148
Median, months	82.2	72.8	78.9
3‐year OS, %	82.4%	75.7%	80.8%

Abbreviations: DFS, disease‐free survival; OS, overall survival.

^a^ Please see Table A1 for details on classification.

## 4 DISCUSSION

Results of this real‐world analysis of patients initially diagnosed with nonmetastatic RCC in the US clinical setting extends our knowledge about risk categories affecting the prognosis for patients. We confirmed the overall prognostic value of tumor‐specific risk classification, as over half of the patients in the population experienced a disease recurrence at some point during post‐nephrectomy follow‐up, and the recurrence rate was higher in the high‐risk cohort as well as among patients with non‐ccRCC/other. The overall 3‐year DFS rate of 49.4% observed in this study, based on a median follow‐up of about 3 years (39.3 months), was similar to the estimated 3‐year DFS reported among postsurgical patients who received placebo in the recent IMmotion010 (53.5%) and KEYNOTE‐564 (62.7%) clinical trials.^23^, ^24^ A previous study in SEER‐Medicare showed a recurrence rate of 41.8% in a similar population.^17^ Such nontrivial recurrence rates observed from varied data sources support the potential benefits of adjuvant treatment capable of preventing or delaying recurrence in early‐stage RCC patients. Finally, our finding of a median OS of almost 79 months aligns with recently published OS results from the KEYNOTE‐564, where median OS had not been reached at 70 months postrandomization.^15^, ^25^

Current and previous data have suggested important links between RCC severity, recurrence, and prognosis in patients with locoregional disease. Our observation of over half the patients in our risk‐enriched study population experiencing a recurrence is consistent with previous findings suggesting that up to 40% of patients with locoregional RCC will recur after surgery, depending on disease stage, severity, and histology, among other factors.^10^, ^11^ Taken together with our other finding that among patients with at least 5 years of follow‐up, recurrence is the most important driver of mortality, this highlights the importance of recurrence prevention after nephrectomy.

Our patient journey assessment showed that most patients underwent nephrectomy after RCC diagnosis without delay, but the data also suggest that there may have been potential care gaps post nephrectomy. However, the gaps noted in this study might be due to missing data as services received outside of participating practices might not have been captured: there was no record of a post‐nephrectomy follow‐up medical oncology visit for one‐third of patients, and only 42% of those who would eventually experience a recurrence attended an oncology visit between nephrectomy and recurrence. Also, fewer than 60% of patients had records of follow‐up imaging post nephrectomy despite the 2014 NCCN Guidelines® recommending imaging every 3–6 months for 2–3 years (based on stage of disease), followed by annual imaging for another 2 years after resection.^26^ Current NCCN Guidelines add that imaging may continue beyond 5 years, if clinically indicated.^9^ Of the imaging records available, most assessments were ordered by medical oncologists, and were most frequently CT scans, followed by MRI and PET imaging. It is likely that medical oncologists ordered these studies as patients with intermediate‐high or high‐risk disease transfer their care from urology to medical oncology after surgery.

As much of the study period predated the availability of approved adjuvant therapies, we were not surprised to see that only 5.9% of patients received systemic adjuvant therapy. Since then, sunitinib and pembrolizumab have been approved by the US FDA (in 2017 and 2021, respectively)^13^, ^14^ and have been incorporated into the NCCN Guidelines as adjuvant treatment options: sunitinib can be prescribed for certain patients with stage III ccRCC, while pembrolizumab is a recommended option for certain patients with stage II‐IV ccRCC.^9^ Sunitinib was approved based on improved DFS versus placebo in a phase 3 clinical trial; however, the treatment was associated with high toxicity and reduced quality of life.^27^ Pembrolizumab became the first immunotherapy approved based on results of the phase 3 KEYNOTE‐564 trial, in which patients with locally advanced or metastatic ccRCC who received pembrolizumab after nephrectomy showed a significant increase in 24‐month DFS versus placebo (HR 0.68 [95% CI: 0.53, 0.87] *p* = 0.002) with no detriment in patient‐reported outcomes.^23^ The tyrosine kinase inhibitors axitinib,^28^ pazopanib and sorafenib,^29^ the mTOR inhibitor everolimus,^30^ and immune checkpoint inhibitors nivolumab, ipilimumab, and atezolizumab^24^, ^31^, ^32^ have also been investigated in this patient population; however, no evidence of meaningful DFS improvements with these agents have been observed to date and almost all were associated with increased toxicity.^8^ As the therapeutic landscape continues to evolve enhanced risk assessment and modeling based on data obtained from this and future real‐world analyses will facilitate improved treatment algorithms.

### 4.1 Limitations

This study shares limitations common to real‐world, retrospective EMR analyses, and the results must be interpreted accordingly. Notably, the data reflect treatment practice patterns limited to US oncologist practices that provide data to the ConcertAI Oncology Dataset. Therefore, imaging assessments, procedures, or visits outside of this network may not have been captured, which could explain missing data for certain variables.

We could not reach conclusions regarding differential outcomes based on tumor grade given the small size of some subgroups. Further, patients within this dataset may differ from the general nonmetastatic RCC population in ways that may not be measurable, and patient sampling procedures may have introduced unmeasurable selection bias, and therefore, may not represent all patients with RCC in the community oncology setting.

## 5 CONCLUSIONS

This study represents the first real‐world analysis of post‐nephrectomy treatment patterns for resected primary RCC patients in the pre‐immunooncology era. Results showed substantial 3‐year recurrence rates among patients with intermediate‐high‐risk and high‐risk RCC, comparable to those reported in KEYNOTE‐564.^23^ Study findings confirm that the prognosis of patients with nonmetastatic RCC is influenced by a range of tumor‐dependent variables, and it is hoped that judicious use of newly approved adjuvant therapies will result in more favorable long‐term outcomes.

## AUTHOR CONTRIBUTIONS

**Jose A. Karam:** Writing – review and editing (equal). **Rituparna Bhattacharya:** Conceptualization (equal); funding acquisition (equal); methodology (equal); writing – review and editing (equal). **Adesuwa Ogbomo:** Investigation (equal); project administration (equal); visualization (equal); writing – review and editing (equal). **Santosh Gautam:** Investigation (equal); supervision (equal); writing – review and editing (equal). **Rebekah Yu:** Investigation (equal); project administration (equal); writing – review and editing (equal). **Murali Sundaram:** Conceptualization (equal); funding acquisition (equal); methodology (equal); writing – review and editing (equal). **Kentaro Imai:** Writing – review and editing (equal). **Jatin Chhabra:** Data curation (equal); formal analysis (equal); writing – review and editing (equal). **Naomi B. Haas:** Writing – review and editing (equal).

## FUNDING INFORMATION

Merck Sharp & Dohme LLC, a subsidiary of Merck & Co., Inc., Rahway, NJ, USA sponsored this study and provided financial support for the conduct of the research and preparation of the article. Merck Sharp & Dohme LLC, a subsidiary of Merck & Co., Inc., Rahway, NJ, USA (the sponsor) collaborated on the design of the study, interpretation of the analyses, and in the decision to submit the article for publication. The sponsor did not have a direct role in data collection or data analysis.

## CONFLICT OF INTEREST STATEMENT

JAK reports honoraria for scientific advisory board/consulting for Merck, Pfizer, Johnson and Johnson; stock ownership in MedTek, ROMTech; and research funding to his institution from Mirati, Roche/Genentech, Merck, and Elypta. RB, MS, and KI are employed by Merck Sharp & Dohme LLC, a subsidiary of Merck & Co., Inc., Rahway, NJ, USA and may hold stock or stock options in Merck & Co., Inc., Rahway, NJ, USA. RY reports research funding to her institution from Merck Sharp & Dohme LLC, a subsidiary of Merck & Co., Inc., Rahway, NJ, USA. AO, SG, and JC were employed at ConcertAI during the conduct of the study. NBH reports participation on advisory boards for Merck, Eisai, Aveo, Exelixis, and Roche.

## INSTITUTIONAL REVIEW BOARD

This research was reviewed and determined to be exempt from Institutional Review Board (IRB) oversight by Advarra IRB (Columbia, Maryland). This research study was conducted retrospectively from data obtained for clinical purposes.

## Data Availability

ConcertAI does not make datasets publicly available because study data are used under license from source practices and other data providers. ConcertAI will consider requests to access study datasets on a case‐by‐case basis. Please contact us with any inquiries at https://www.concertai.com/contact‐us/.

## APPENDIX 1

**TABLE A1:** Classification of renal cell carcinomas.

Clear‐cell carcinoma	Nonclear‐cell carcinoma	Other histology types (Other)
Clear cell adenocarcinoma	Acquired cystic disease associated renal cell carcinoma	Carcinoma, NOS
Clear cell adenocarcinoma, NOS	Chromophobe renal cell carcinoma	Clear cell papillary renal cell carcinoma
Clear cell renal cell carcinoma	Chromophobe renal cell carcinoma/renal cell carcinoma, sarcomatoid	Clear cell papillary renal cell carcinoma/renal cell carcinoma, sarcomatoid
Clear cell renal cell carcinoma/Renal cell carcinoma, sarcomatoid	Collecting duct carcinoma	Clear cell renal cell carcinoma/papillary renal cell carcinoma
	Papillary adenocarcinoma, NOS	Mixed cell adenocarcinoma
	Papillary carcinoma, NOS	Mucinous adenocarcinoma
	Papillary renal cell carcinoma	Papillary renal cell carcinoma/renal cell carcinoma, sarcomatoid
	Renal cell carcinoma, chromophobe type	Renal cell carcinoma, NOS
		Renal cell carcinoma, NOS/renal cell carcinoma, sarcomatoid
		Renal cell carcinoma, chromophobe type/renal cell carcinoma, sarcomatoid
		Renal cell carcinoma, sarcomatoid
		Transitional cell carcinoma, NOS

Abbreviation: NOS, not otherwise specified.

**TABLE A2:** Treatment regimens, by RCC diagnosis year and risk category.

Year of RCC Diagnosis	Regimen Number	Regimen	Intermediate‐High Risk (*N* = 377) (*n* = 21)	High Risk (*N* = 62) (*n* = 5)	Overall (*N* = 439) (*n* = 26)
2012	1	Everolimus	1 (4.76%)	0 (0.0%)	1 (3.85%)
	1	Letrozole	1 (4.76%)	0 (0.0%)	1 (3.85%)
	1	Sunitinib	1 (4.76%)	0 (0.0%)	1 (3.85%)
	1	Temsirolimus	0 (0.0%)	1 (20.00%)	1 (3.85%)
2013	1	Sunitinib	3 (14.29%)	0 (0.0%)	3 (11.54%)
2014	1	Pazopanib	1 (4.76%)	0 (0.0%)	1 (3.85%)
2015	1	Pazopanib	1 (4.76%)	1 (20.00%)	2 (7.69%)
2016	1	Pazopanib	4 (19.05%)	0 (0.0%)	4 (15.38%)
	1	Sunitinib	1 (4.76%)	1 (20.00%)	2 (7.69%)
	1	Denosumab, pazopanib	1 (4.76%)	0 (0.0%)	1 (3.85%)
	2	Sunitinib	0 (0.0%)	1 (20.00%)	1 (3.85%)
2017	1	Pazopanib	1 (4.76%)	2 (40.00%)	3 (11.54%)
	1	Sunitinib	3 (14.29%)	0 (0.0%)	3 (11.54%)
	1	Bortezomib	1 (4.76%)	0 (0.0%)	1 (3.85%)
	1	Cyclophosphamide, doxorubicin, rituximab, vincristine	1 (4.76%)	0 (0.0%)	1 (3.85%)
	1	Ipilimumab, nivolumab	1 (4.76%)	0 (0.0%)	1 (3.85%)
	2	Carfilzomib	1 (4.76%)	0 (0.0%)	1 (3.85%)
	2	Pazopanib	0 (0.0%)	1 (20.00%)	1 (3.85%)

Abbreviation: RCC, renal cell carcinoma.

**TABLE A3:** First visit versus first date of recurrence.

Patients with Recurrence (*N* = 223)	*n*/%
1. Patients with visit before recurrence date	
*n* (%)	93 (41.7%)
Mean # of visits (*n*) before first recurrence	17.7
Median # of visits (*n*) before first recurrence	4.0
2. Patients with visit on or after recurrence date	
*n* (%)	189 (84.8%)
Mean # of visits (*n*) on or after recurrence	23.4
Median # of visits (*n*) on or after recurrence	11.0
3. Patients with visit before and after recurrence date	
*n* (%)	93 (41.7%)
Mean # of visits (*n*) throughout follow‐up period	32.0
Median # of visits (*n*) throughout follow‐up period	17.0

*Note*: There were 38 patients with no record of a visit before or after their first recurrence. Groups are not mutually exclusive, i.e., patients with visit before and after recurrence (3) may also be included in either (1) or (2).
